# Is there loss or qualitative changes in the expression of thyroid peroxidase protein in thyroid epithelial cancer?

**DOI:** 10.1054/bjoc.2001.2015

**Published:** 2001-09

**Authors:** B Czarnocka, D Pastuszko, M Janota-Bzowski, A P Weetman, P F Watson, E H Kemp, R S McIntosh, M S Asghar, B Jarzab, E Gubala, J Wloch, D Lange

**Affiliations:** Department of Biochemistry, Medical Center of Postgraduate Education, Marymoncka 99, Warsaw, 01–813, Poland; Department of Endocrinology, Medical Research Center, Polish Academy of Science, Warsaw, Poland; Department of Medicine, University of Sheffield Clinical Sciences Center, Northern General Hospital, Sheffield, S5 7AU, United Kingdom; Clinical Immunology Unit, Queen's Medical Center, Nottingham, NG7 2UH, United Kingdom; Oncology Center, M Sklodowska-Curie Memorial Institute, Gliwice, Poland

**Keywords:** differentiated thyroid cancer, TPO, mAb, IgGκ, Fab, epitope

## Abstract

There is disagreement concerning the expression of thyroid peroxidase (TPO) in thyroid cancer, some studies finding qualitative as well as quantitative differences compared to normal tissue. To investigate TPO protein expression and its antigenic properties, TPO was captured from a solubilizate of thyroid microsomes by a panel of murine anti-TPO monoclonal antibodies and detected with a panel of anti-human TPO IgGκ Fab. TPO protein expression in 30 samples of malignant thyroid tissue was compared with TPO from adjacent normal tissues. Virtual absence of TPO expression was observed in 8 cases. In the remaining 22 malignant thyroid tumours the TPO protein level varied considerably from normal to nearly absent when compared to normal thyroid tissue or tissues from patients with Graves' disease (range less than 0.5 to more than 12.5 μg mg^−1^ of protein). When expressed TPO displayed similar epitopes, to that of TPO from Graves' disease tissue. The results obtained by the TPO capturing method were confirmed by SDS-PAGE and Western blot analysis with both microsomes and their solubilizates. The present results show that in about two-thirds of differentiated thyroid carcinomas, TPO protein is expressed, albeit to a more variable extent than normal; when present, TPO in malignant tissues is immunologically normal. © 2001 Cancer Research Campaignhttp://www.bjcancer.com


British Journal of Cancer (2001) 85(6), 875–880
© 2001 Cancer Research Campaign
doi: 10.1054/ bjoc.2001.2015, available online at http://www.idealibrary.com on IDEAL

http://www.bjcancer.com

### Is there loss or qualitative changes in the expression of thyroid peroxidase protein in thyroid epithelial cancer?

**B Czarnocka[1,2], D Pastuszko[1], M Janota-Bzowski[1], AP Weetman[3], PF Watson[3], EH Kemp[3], RS McIntosh[4], MS Asghar[4], B Jarzab[5], E Gubala[5], J Wloch[5] and D Lange[5]**

[1]Department of Biochemistry, Medical Center of Postgraduate Education, Marymoncka 99,01–813 Warsaw, Poland; [2]Department of Endocrinology, Medical Research Center, Polish Academy of Science, Warsaw, Poland; [3]Department of Medicine, University of Sheffield Clinical Sciences Center, Northern General Hospital, Sheffield, United Kingdom S5 7AU; [4]Clinical Immunology Unit, Queen's Medical Center, Nottingham, United Kingdom NG7 2UH; [5]Oncology Center, M Sklodowska-Curie Memorial Institute, Gliwice, Poland

**Summary** There is disagreement concerning the expression of thyroid peroxidase (TPO) in thyroid cancer, some studies finding qualitative as well as quantitative differences compared to normal tissue. To investigate TPO protein expression and its antigenic properties, TPO was captured from a solubilizate of thyroid microsomes by a panel of murine anti-TPO monoclonal antibodies and detected with a panel of anti-human TPO IgGκ Fab. TPO protein expression in 30 samples of malignant thyroid tissue was compared with TPO from adjacent normal tissues. Virtual absence of TPO expression was observed in 8 cases. In the remaining 22 malignant thyroid tumours the TPO protein level varied considerably from normal to nearly absent when compared to normal thyroid tissue or tissues from patients with Graves' disease (range less than 0.5 to more than 12.5 μg $mg^{-1}$ of protein). When expressed TPO displayed similar epitopes, to that of TPO from Graves' disease tissue. The results obtained by the TPO capturing method were confirmed by SDS-PAGE and Western blot analysis with both microsomes and their solubilizates. The present results show that in about two-thirds of differentiated thyroid carcinomas, TPO protein is expressed, albeit to a more variable extent than normal; when present, TPO in malignant tissues is immunologically normal. © 2001 Cancer Research Campaign http://www.bjcancer.com

**Keywords**: differentiated thyroid cancer; TPO; mAb; IgGκ Fab; epitope

Papillary (PTC) and follicular thyroid carcinomas (FTC) are usually well differentiated, maintaining in part the characteristics of follicular thyroid cells, including expression of thyroid cell-specific antigens such as thyroid peroxidase (TPO), the membrane-bound enzyme essential for the biosynthesis of thyroid hormone. Recently, TPO gene and protein expression in thyroid carcinoma has been analysed, with results indicating low enzymatic activity (Valenta et al, 1973; Valenta, 1976; Fragu and Nataf, 1977; Mizukami and Matsunaga, 1981), and impaired solubility (Neary et al, 1978). Mutated TPO genes were found in some differentiated thyroid carcinomas (Smanik et al, 1994). Although the expression of TPO mRNA was found to be the same in cancerous and benign tissue in one study (Ohta et al, 1991), others have shown suppression of TPO gene expression (Hoang-Vu et al, 1992; Fabbro et al, 1994; Tanaka et al, 1996; Umeki et al, 1996).

Some differences in the results of experiments using TPO murine monoclonal antibodies to detect protein expression may be due to methodological approaches (Yamashita et al, 1993; Mizukami et al, 1994; Tanaka et al, 1996; Umeki et al, 1996). However, differential immunohistochemical labelling of TPO with one monoclonal antibody (mAb#47) was proposed by De Micco et al (1991) as a marker distinguishing between benign and malignant thyroid tumours, including both PTC and FTC. Reduced immunostaining with mAb#47 was observed in more than 95% of malignant thyroid cancers suggesting that abnormal TPO immunoreactivity occurs in malignancy (DeMicco et al, 1991, 1994). MAb #47 is one of a panel of well characterized murine monoclonal antibodies produced against native human TPO. The immunochemical properties of these mAbs have been published elsewhere (Ruf et al, 1989).

In order to clarify whether TPO in thyroid cancers is indeed qualitatively different from TPO in non-cancerous tissues, we analysed the reactivity of TPO from thyroid cancers with a panel of murine monoclonal antibodies (including mAb#47) and previously described human monoclonal anti-TPO IgGκ Fab fragments (McIntosh et al, 1997). Both types of antibodies react with epitopes delineating the immunodominant region of TPO (Figure 1).

### METHODS

### Thyroid tissues

We studied 30 malignant thyroid tumours, 23 of them paired with corresponding normal tissue obtained from adjacent parts of the gland. Thyroid tissues were obtained from patients undergoing surgery for thyroid cancer by the Institute of Oncology, Gliwice, Poland. Tissues fragments were immediately frozen in liquid $N_2$, and kept at –80˚C until use. Tumours were diagnosed histopathologically according to World Health Organization (WHO) criteria: 3 cases were follicular carcinomas (FTC) and 27 cases were papillary carcinomas (PTC). 10 thyroid samples from patients with Graves' disease were also included in the study. Details of the patients are summarized in Table 1. The local Committees on

*Received 26 October 2000*
*Revised 11 June 2001*
*Accepted 2 July 2001*

*Correspondence to:* B Czarnocka

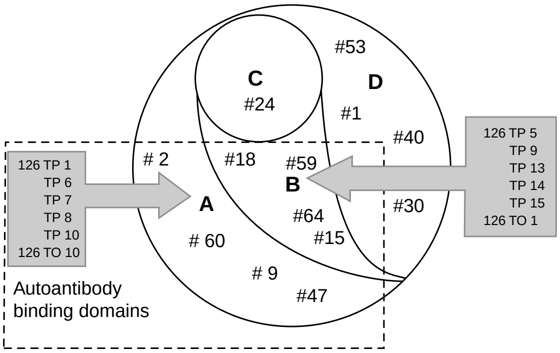

**Figure 1** Map of the epitopes on thyroid peroxidase (TPO) recognized by murine monoclonal antibodies (mAb) and recombinant human TPO specific Fabs

Medical Ethics approved these studies and all patients gave their written consent.

### Murine mAb and human recombinant IgGκ antibodies

A panel of 12 murine anti-TPO monoclonal antibodies (mAb), previously produced and characterized, directed to epitopes from 3 antigenic domains of TPO was used (Ruf et al, 1989). Antibodies mAb#2, #9, #47, #60 – recognize domain A; mAb#15, #18, #59, #64 – domain B, and mAb#1, #30, #40, #53 – domain D. Domains A and B, defining the immunodominant region of TPO, are adjacent whereas domain D is topologically distant. Characterization of the human Fabs has been described elsewhere (Czarnocka et al, 1997; McIntosh et al, 1997). In this study, Fabs representative for domain A (126 TP1, 6, 7, 8, 10 and 126 TO 10), and domain B reactivity (126 TP5, 9, 13, 14, 15 and 126 TO1) were used. All experiments were carried out on Fab prepared in culture medium.

### Thyroid microsomes preparation and solubilization

Thyroid microsomes were prepared and solubilized as previously described (Czarnocka et al, 1985). Briefly, the thyroid tissues were homogenized in 10 mM Tris-HCl buffer pH 7.5, containing 2 μg ml$^{-1}$ aprotinin, 1 μg ml$^{-1}$ leupeptin, 1 μg ml$^{-1}$ pepstatin and 0.1 mM phenylmethylsulfonyl fluoride (all from Boehringher Mannheim, Mannheim, Germany). The plasma membranes were pelleted by centrifugation at 10 000 ***g*** for 15 min at 4˚C and the supernatants were subsequently ultracentrifuged at 100 000 ***g*** for 60 min at 4˚C. The resulting membrane fractions were solubilized with sodium deoxycholate, and stored at –80˚C until use. The protein concentration was determined by the BCA micro-method (Pierce Chemical Co, Rockford, IL, USA).

### TPO capture method

The antigen capture method was used to detect solubilized TPO. Maxisorb microtitre plates (Nunc, Copenhagen, Denmark) were coated with 100 μl of mAb solution diluted to 10 μg ml$^{-1}$ in 0.1 M carbonate buffer pH 9.6, overnight at 4˚C under a humidified atmosphere. The wells were then washed, blocked with BSA, washed again, and filled with 100 μl of solubilizate adjusted to 20 μg ml$^{-1}$ in PBS pH 7.8, containing 0.1% of Tween-20 and 3 mM sodium azide. After overnight incubation at 4˚C, unbound protein was removed by extensive washing and wells filled with 100 μl of

**Table 1** Clinical data

| Patient no | Sex/age (y) | Histology | pTNM | TSH |
|---|---|---|---|---|
| 1 | M/54 | PTC + NT | $T_{3a}$ | NA |
| 2 | F/65 | FTC + NT | NA | 0,15 |
| 3 | F/43 | PTC + NT | $T_2$ | 3,43 |
| 4 | F/60 | FTC + NT | $T_{2b}$ | 4,88 |
| 5 | F/50 | PTC + NT | $T_2$ | 1,12 |
| 6 | M/45 | PTC | $T_{2b}$; $N_1$ | NA |
| 7 | F/64 | PTC | $T_3$ | NA |
| 8 | F/24 | PTC + NT | $T_{2b;}$; $N_1$ | 0,7 |
| 9 | F/65 | PTC + NT | NA | NA |
| 10 | F/46 | PTC + NT | $T_{1a}$ | 0,4 |
| 11 | F/59 | PTC | $T_1$ | 10,0 |
| 12 | F/39 | PTC + NT | $T_{1a}$ | 0,91 |
| 13 | F/28 | PTC | $T_{2b}$ | 1,18 |
| 14 | M/27 | PTC | $T_3$ | 0,69 |
| 15 | F/48 | PTC + NT | $T_{2b}$ | 0,28 |
| 16 | M/22 | PTC + NT | $T_1$; $N_1$ | 0,99 |
| 17 | F/55 | PTC + NT | $T_3$ | 7,60 |
| 18 | F/51 | PTC + NT | $T1_a$ | NA |
| 19 | M/21 | PTC + NT | $T_4$; $N_{1b}$ | NA |
| 20 | F/61 | PTC + NT | $T_2$; $N_1$ | NA |
| 21 | M/35 | PTC + NT | NA | NA |
| 22 | M/31 | PTC | $T_2$; $N_1$ | NA |
| 23 | F/47 | PTC + NT | $T_2$ | 1,47 |
| 24 | F/62 | FTC + NT | $T_{2b}$ | 1,55 |
| 25 | F/16 | PTC + NT | $T_{2b}$ | NA |
| 26 | F/17 | PTC + NT | $T_2$ | NA |
| 27 | F/43 | PTC + NT | $T_{1a}$; $N_1$ | NA |
| 28 | M/32 | PTC | NA | NA |
| 29 | M/65 | PTC + NT | $T_{2b}$; $N_1$ | 0,89 |
| 30 | F/38 | PTC + NT | $T_2$ | 1,12 |
| 31, 32 | F/56, F/47 | Graves' disease | | |
| 33, 34 | F/62, F/58 | Graves' disease | | |
| 35, 36 | F/67, M/55 | Graves' disease | | |
| 37, 38 | F/50, M/60 | Graves' disease | | |
| 39, 40 | F/42, F/39 | Graves' disease | | |

T = primary tumour; N = lymph node metastasis; M = metastasis; NA = not available; tumour size: $T_1$ < 1 cm; $T_2$ 1–5 cm; $T_3$ > 5 cm (tumour without penetration of thyroid capsule); $T_4$ = infiltration through thyroid capsule); $T_a$ = monocentric; $T_b$ = multicentric. $N_0$ = tumour without metastasis to lymph nodes; $N_1$ = metastasis to lymph nodes; $N_{1a}$ = ipsilateral metastasis; $N_{1b}$ = contralateral metastasis; $M_0$ = without distant metastasis; $M_1$ = distant metastasis.

Fab diluted 1:15 with PBS-Tween-20-azide. After 12 h at 4˚C, unbound antibodies were removed by extensive washing. Fab binding was detected using an immunoaffinity-purified antihuman Fab second antibody labelled with horseradish peroxidase (Jackson ImmunoResearch Laboratories, Inc, West Grove, PA, USA); tetramethyl benzidine was the substrate. Absorbance was read at 450 nm.

### SDS-PAGE and Western blot

Thyroid microsomes as well as their solubilizates were diluted with buffer containing 0.125 M Tris-HCl pH 6.8, 10% β-mercaptoethanol, 4% SDS, and 20% glycerol, to a concentration of 1 mg ml$^{-1}$, boiled and loaded (20 μg lane$^{-1}$) onto 10% SDS polyacrylamide mini gels. Non-denaturing sample buffer consisted of 0.3125 M Tris, 0.5 M dithiothreitol, 1.5% SDS and 50% glycerol and microsomes or solubilizates were diluted to 1 mg ml$^{-1}$ with the above buffer and incubated for 30 min at 37˚C and loaded (20 μg lane$^{-1}$) onto SDS polyacrylamide mini gels, 0.75 mm thick. Proteins were

 *© 2001 Cancer Research Campaign*

directly electro-transferred onto Immobilon P (Millipore, Bedford Corp, MA, USA), and these membranes were then blocked with 3% BSA in PBS-3 mM sodium azide for 60 min at room temperature. Western blots were done by using a 1:500 dilution of anti-TPO mAb#47 antibody or a 1:1000 dilution anti-TPO rabbit polyclonal antibody in PBS-0.1% Tween 20 with 3 mM sodium azide overnight at 4˚C with constant shaking. After washing 3 times for 15 minute in PBS-Tween 20, membranes were incubated for 60 min at room temperature under shaking with either immunoaffinity-purified antimouse IgG conjugated to horseradish peroxidase or immunoaffinity-purified antirabbit IgG conjugated to horseradish peroxidase (Jackson ImmunoResearch, Laboratories, Inc, West Grove, PA, USA). After additional 3 washes, the blots were developed with DAB – metal enhanced, precipitating (Boehringer Mannheim, Mannheim, Germany) as substrate.

### RESULTS

### TPO capture

We observed 3 patterns of TPO protein expression. In 8 malignant tumours the TPO level was very low or undetectable, in 19 samples there was a variable TPO protein level from very low to almost normal and in 3 there was a high TPO level. Figure 2 shows the pattern of Fab binding to TPO captured by mAbs in tissues representative of these 3 groups, adjacent normal tissue and, as a control, Graves' thyroid.

To assess the relative amount of TPO protein expressed in thyroid cancer tissues, the results of captured highly purified TPO at concentrations 1.0, 5.0, 10.0 and 25.0 ng well$^{-1}$ were compared to the data obtained for cancer tissues. The relative concentration of TPO in the 30 tumours ranged from much less than 0.5 μg mg$^{-1}$ in 8 cases to more than 12.5 μg mg$^{-1}$ of the protein in 3 cases. In the majority of cancerous tissues however, the TPO amount estimated by the method used ranged from 2.5 to 5 μg mg$^{-1}$ of microsomes protein (data not shown).

### Epitope mapping

To get a better insight in the characteristics of the TPO antigenic activity we analysed the relationship between Fab binding to the TPO and the mAbs used for the antigen capture. We observed a cross-competitive effect between mAbs and IgGκ Fab for the binding sites on the TPO molecule in all cases studied. The Fab binding to TPO showed cross-reactive inhibition dependent on which mAb was used for TPO capture. A positive relationship of the Fab binding to TPO was observed, especially when TPO was captured by mAbs from domain B. The binding of all Fabs reactive to domain A (126 TP 1, TP 6, TP 7, TP 8, TP 10 and 126 TO 10) was high, whereas the binding of Fabs reacting with epitopes located on domain B (126 TP 5, TP 9, TP 13, TP 14, TP 15 and 126 TO 1) was substantially reduced.

Strong inhibition of the binding of Fabs reacting with epitopes located on antigenic domain B with TPO immobilized by domain B mAbs indicated that epitopes for both mAbs and Fabs were overlapping. This effect was less pronounced when TPO was bound to mAbs from antigenic domain A. Fabs reacting with epitopes located on antigenic domain A react predominantly with antigenic domain A with some cross-reactivity to domain B. When TPO was bound to mAbs from antigenic domain D (Figure 2) no competition between mAbs and Fabs was observed. All Fabs, whether reactive with antigenic domains A or B, bound equally, indicating that the epitopes of these monoclonal antibodies and the Fab-binding sites did not overlap each other. The observed differences in absorbance values were due to variations in Fab affinity for the TPO.

The same pattern of reactivity was observed when TPO from normal and Graves' thyroid tissues was captured (see Figure 2B and 2F). However, the cross-competitive effects between mAbs and Fabs for binding sites on the TPO was less apparent due to the high quantity of TPO present in hyperfunctioning tissue.

### SDS-PAGE and Western blot analysis

In order to confirm the presence or absence of TPO in cancerous and normal tissues observed using the antigen capture method, TPO was analysed by SDS-PAGE and Western blot. The intensity of immunostaining of the TPO bands correlated well with the TPO level detected by the antigen capture method (Figure 3A, B). In normal and cancerous tissues taken from the same individuals, TPO bands were highly immunoreactive and well visualized in the normal, but not in the corresponding cancerous tissue (Figure 3B). Only 8 of 30 cancer tissues were negative in Western blot with both antibodies (Figure 3A; patients 10 and 11). In the remaining 22 cancer tissues, the TPO band intensity varied from very slight to strong immunostaining with mAb#47 as well as with rabbit polyclonal anti-TPO antibodies. Using identical amounts of protein allowed measurement of the relative quantity of the TPO with respect to intensity and size of the TPO band (Figure 3C). Densitometric evaluation of the blots demonstrated that in cancerous tissues, the amount of TPO was 3–20 times lower than in paired normal tissues.

### DISCUSSION

In the first part of this study, we evaluated the presence or absence of TPO protein in differentiated thyroid carcinomas and estimated the approximative amount of the TPO expressed. Solubilized TPO was captured by the same panel of anti-TPO murine monoclonal antibodies used by De Micco et al (1991). TPO was undetectable or barely detected in 8 of 30 differentiated carcinomas as compared with the normal paired thyroid tissues. Variable TPO protein content was found in 19 cancer tissues, and in 3 tissues TPO was present at a high level, comparable to that detected in the corresponding normal or Graves' disease tissues. Several groups have found that one-third of papillary thyroid carcinomas studied do not express TPO mRNA, and thus lack TPO enzyme (Hoang-vu et al, 1992; Fabbro et al, 1994; Tanaka et al, 1996). These cases should not be immunoreactive with either mAbs or Fabs. The frequency (26.7%) of differentiated thyroid carcinomas with negative or extremely low TPO level found in our study (albeit with 6 of them still showing a trace of TPO protein by the method used) is therefore similar to these reports. Others have produced contradictory data, demonstrating similar if not identical TPO mRNA levels in a series of malignant thyroid tumours and in benign tissues (Ohta et al, 1991) for reasons which are not clear.

The level of TPO detected in cancer tissues varied considerably from normal to virtually absent. Considering the limitations of antigen capture as a quantitative method, we could only estimate the relative amount of the TPO present in cancerous tissues, which

*© 2001 Cancer Research Campaign* 

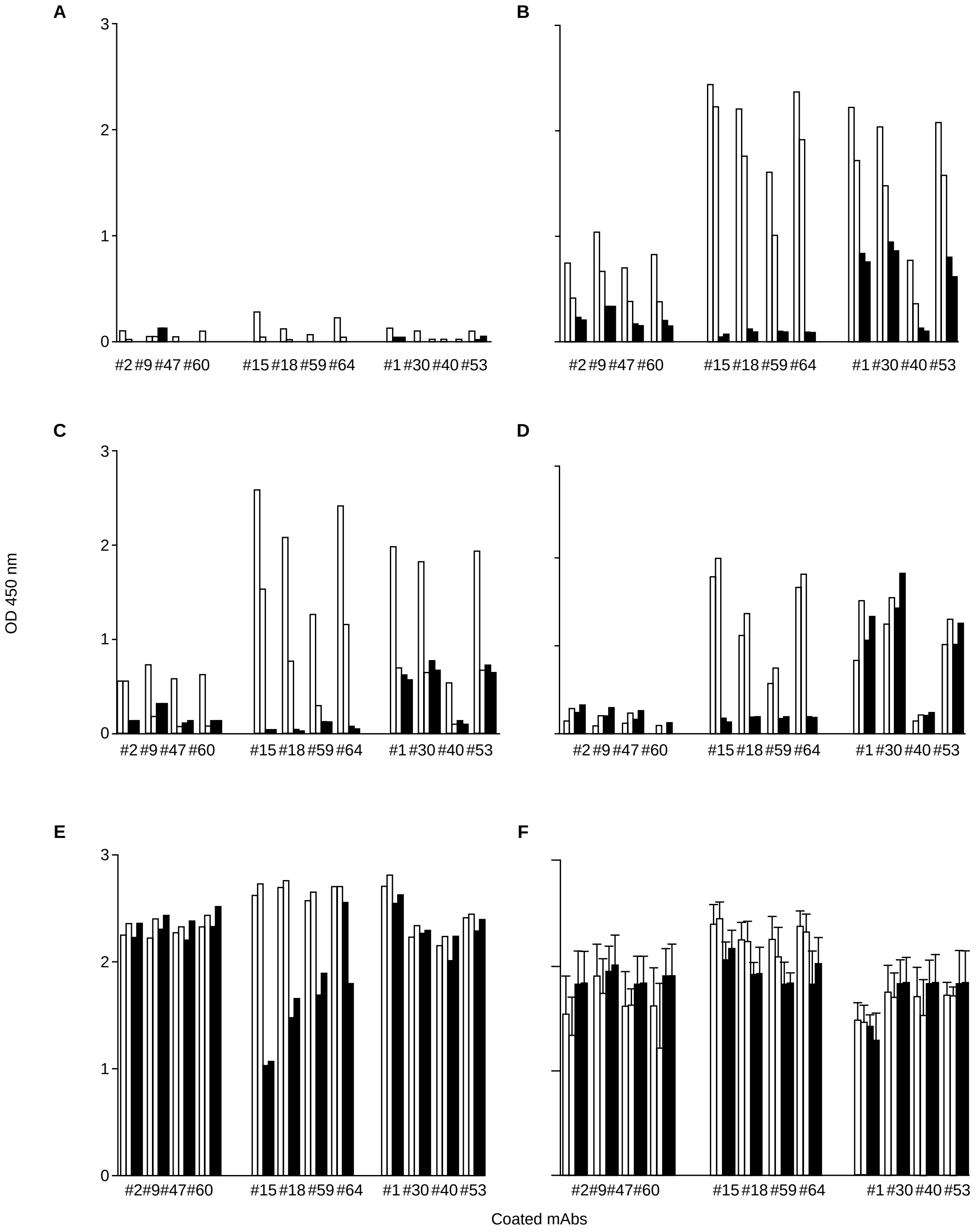

**Figure 2** The results of Fab binding to TPO captured by a panel of mAbs in representative cancer tissues. Open bars: Fabs 126 TP1 and 126TP7, reactive to domain A – (identical patterns were observed for Fabs 126TP6, TP8, TP10 and 126 TO10). Filled bars: Fabs 126TP5 and 126TP15, reactive to domain B – (identical patterns were observed for Fabs 126TP 9, TP 13, TP14 and 126TO1). (**A**) representative results for tumours with very low TPO level; (**B**) paired normal tissue; (**C, D**) representative results for tissues expressing variable TPO protein level; (**E**) the data of one of 3 cases with a high TPO level expression; and (**F**) the results obtained for control Graves' tissue (for Graves' thyroid tissues experiments data presented are mean ± SD). Solid phase coated mAb were used for TPO capture and Fab in solution used for TPO detection. See Material and Methods for experimental details ranged from much less than 0.5 μg to more than 12.5 μg $mg^{-1}$ of microsome proteins, but the majority was around 2.5 μg $mg^{-1}$ to 5 μg $mg^{-1}$ of the protein. The presence or absence of TPO in cancerous tissues detected in antigen capture method was confirmed by SDS-PAGE and Western blot. The immunolabelling of the TPO bands with mAb#47 demonstrate that mAb#47 epitope was expressed on the TPO molecule. Immunohistochemical techniques using monoclonal antibodies generated against native

*© 2001 Cancer Research Campaign*

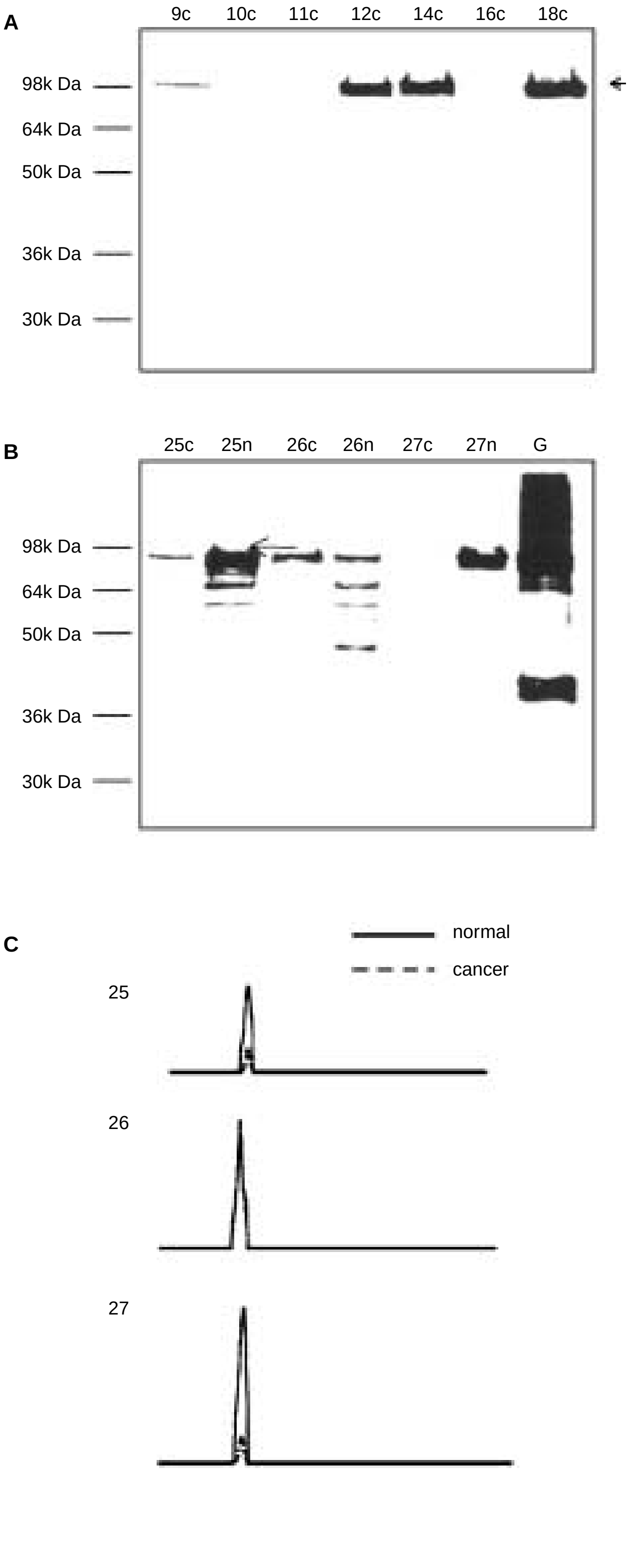

**Figure 3** Western blot analysis of TPO in thyroid cancer tissue after SDS-PAGE separation of microsome proteins (20 μg lane$^{-1}$) under reducing conditions probed with anti-TPO mAb#47. (**A**) Results representative of cancer tissues; (**B**) 3 representative cancer tissues and corresponding normal thyroid tissue; (**C**) scannergrams comparing the density of the TPO bands shown in (**B**). The position of TPO bands are shown by arrows; the molecular masses of standards are on the left. Papillary thyroid carcinoma (c), corresponding normal paired tissue (n), Graves' disease thyroid tissue (G).

human TPO have also been frequently used to demonstrate variable levels of TPO protein in thyroid follicular cells under various pathological conditions, including a large series of malignant thyroid tumours (DeMicco et al, 1991; Yamashita et al, 1993; Mizukami et al, 1994; Tanaka et al, 1996). The results of immunohistochemical methods also differ substantially. Using immunostaining with 2 (mAb#30 and mAb#47) of 13 mAbs against native human TPO, De Micco et al (1991) demonstrated differences in TPO immunoreactivity between benign and malignant tumours and, on this basis, mAb#47 has been proposed as a specific marker of malignancy. These and other data reported by this group suggest that although the TPO molecule is present in thyroid cancers at low levels, the epitope recognized by mAb#47 is not (DeMicco et al, 1991, 1994; Faroux et al, 1997; Garcia et al, 1998).

To detect any modifications or abnormalities of the TPO molecule expressed in thyroid carcinomas, we chose a structural approach of mapping the antigenic surface with a panel of 13 well characterized mAbs directed against native human TPO, and a panel of human anti-TPO IgGκ Fab fragments (Ruf et al, 1989; McIntosh et al, 1997). TPO purified from Graves' tissues was previously used to map the interaction of a panel of 13 mouse mAbs to 4 antigenic domains of TPO (Ruf et al, 1989). Human monoclonal autoantibodies expressed as IgGκ Fab fragments recognized exclusively epitopes located in only 2 of the 4 domains, A and B (Czarnocka et al, 1997). Moreover, there was no overlap between the epitopes recognized by the panel of TPO-specific IgGκ Fab and the epitopes of mAbs reacting with antigenic domains C and D (Czarnocka et al, 1997). We observed that the TPO protein present in cancer tissues did not show any differences from normal in its antigenic activity, as TPO was captured by all murine anti-TPO monoclonal antibodies used. Immobilized TPO showed similar reactivity towards human IgGκ Fabs, with clearly pronounced steric hindrance dependent on which mAb was used to capture the TPO.

The occupancy of the TPO surface by the mAbs reactive to antigenic domains A or B inhibited the reactivity of the Fabs with captured TPO. This was clearly seen when TPO was bound via mAbs directed to epitopes located on antigenic domain B, indicating that both mAbs and Fabs recognize overlapping part of TPO. No cross-inhibition of Fab binding to TPO was observed when TPO was captured by mAbs reactive with antigenic domain D. This TPO region is not recognized by autoantibodies, and none of the binding of the recombinant human IgGκ Fabs used was influenced by the occupancy of those mAb epitopes on the TPO molecule (Czarnocka et al, 1997; McIntosh et al, 1997). This result therefore indicates that, when expressed, TPO in differentiated thyroid cancer tissues does not differ from TPO in normal thyroid tissue or Graves' tissue. TPO in the majority of the cancer tissues studied was also efficiently captured by mAb#47 with the exception of the 8 negative cases. From our present study, it is evident that although the TPO protein in the majority of differentiated thyroid cancer cases is lower than normal it has unchanged antigenic activity, when compared to that observed in normal and Graves' disease tissues. Recently, the correlation of TPO immunostaining by mAb#47 with the differentiation and proliferative potential of follicular tumours was examined and a significant correlation between TPO mAb#47 staining and the proliferating cell nuclear antigen index with malignancy was found, suggesting that an alteration of TPO antigenicity is an early marker of thyroid follicular tumours, closely related to tumour growth in the first stages o malignant transformation (Garcia et al, 1998). We found in our series of malignant thyroid carcinoma tissues a graded expression of TPO protein from negative to almost normal. However, regardless of any differences in TPO content, its activity towards both mAbs and Fabs was similar to observed in normal or Graves' disease tissues.

### ACKNOWLEDGEMENTS

This study was supported by Medical Center of Postgraduate Education No CMKP-501-2-1-01-01/98 grant to BC, DP, MJ-B.

### REFERENCES

Czarnocka B, Ruf J, Ferrand M, Lissitzky S and Carayon P (1985) Purification of the human thyroid peroxidase and its identification as the microsomal antigen involved in autoimmune thyroid diseases. *FEBS Lett* **190**: 147–152

Czarnocka B, Janota-Bzowski M, McIntosh RS, Suhail Asghar M, Watson PF, Kemp HE, Carayon P and Weetman AP (1997) Immunoglobulin Gκ antithyroid peroxidase antibodies in Hashimoto's thyroiditis: epitope-mapping analysis. *J Clin Endocrinol Metab* **82**: 2639–2644

De Micco C, Ruf J, Chrestian MA, Gros N, Henry JF and Carayon P (1991) Immunohistochemical study of thyroid peroxidase in normal, hyperplastic, and neoplastic human thyroid tissues. *Cancer* **67**: 3036–3041

De Micco C, Zoro P, Garcia S, Skoog L, Tani EM, Carayon P and Henry J-F (1994) Thyroid peroxidase detection as a tool to assist diagnosis of thyroid nodules on fine-needle aspiration biopsy. *Eur J Endocrinol* **131**: 474–479

Fabbro D, Di Loreto C, Beltorami CA, Belfiore A, Di Lauro R and Damante G (1994) Expression of thyroid-specific transcription factors TTF-1 and Pax-8 in human thyroid neoplasm. *Cancer Res* **54**: 4744–4749

Faroux MJ, Theobald S, Pluot M, Patey M and Menzies D (1997) Evaluation of the monoclonal antibody anti-thyroperoxidase MoAb47 in the diagnostic decision of cold thyroid nodules by fine-needle aspiration. *Pathol Res Pract* **193**: 705–712

Fragu PN and Nataf BM (1977) Human thyroid peroxidase activity in benign and malignant thyroid disorders. *J Clin Endocrinol Metab* **45**: 1089–1096

Garcia S, Vassko V, Hanry J-F and De Micco C (1998) Comparison of thyroid peroxidase expression with cellular proliferation in thyroid follicular tumors. *Thyroid* **9**: 745–749

Hoang-Vu C, Dralle H, Scheumann G, Horn R, von zur Mühlen A and Brabant G (1992) Gene expression of differentiation and dedifferentiation markers in normal and malignant human thyroid tissues. *Exp Clin Endocrinol* **100**: 51–56

Mizukami Y and Matsunaga F (1981) Correlation between thyroid peroxidase activity and histopathological and ultrastructural changes in various thyroid diseases. *Endocrinol Jpn* **28**: 381–389

Mizukami Y, Nonomura A, Michigishi T, Noguchi M, Nakamura S, Arai Y, Kotani T, Ohtaki S and Matsukawa S (1994) Immunohistochemical demonstration of thyroid peroxidase (TPO) in human thyroid tissues from various thyroid diseases. *Anticancer Res* **14**: 1329–1334

McIntosh RS, Asghar MS and Kemp EH (1997) Analysis of immuno-globulin Gκ antithyroid peroxidase antibodies from different tissues in Hashimoto's thyroiditis. *J Clin Endocrinol Metab* **82**: 3818–3825

Neary JT, Nakamura C, Davidson B, Soodak M, Vickery AL and Maloof F (1978) Studies on the membrane-associated nature of human thyroid peroxidase: a difference in the solubility of the enzyme from benign and malignant thyroid tissues. *J Clin Endocrinol Metab* **46**: 791–800

Ohta K, Endo T and Onaya T (1991) The mRNA levels of thyrotropin receptor, thyroglobulin and thyroid peroxidase in neoplastic human thyroid tissues. *Biochem Biophys Res Commun* **174**: 1148–1153

Ruf J, Toubert M-E, Czarnocka B, Durand-Gorde J-M, Ferrand M and Carayon P (1989) Relationship between immunological structure and biochemical properties of human thyroid peroxidase. *Endocrinology* **125**: 1211–1218

Smanik PA, Fithian LJ and Jhiang SM (1994) Thyroid peroxidase expression and DNA polymorphism in thyroid cancer. *Biochem Biophys Res Commun* **198**: 948–954

Tanaka T, Umeki K, Yamamoto I, Sugiyama S, Noguchi S and Ohtaki S (1996) Immunohistochemical loss of thyroid peroxidase in papillary thyroid carcinoma: strong suppression of peroxidase gene expression. *J Pathol* **179**: 89–94

Umeki K, Tanaka T, Yamamoto I, Aratake Y, Kotani T, Sakamoto F, Noguchi S and Ohatki S (1996) Differential expression of dipeptidyl peptidase IV (CD26) and thyroid peroxidase in neoplastic thyroid tissues. *J Endocrine* **43**: 53–60

Valenta LJ (1976) Thyroid peroxidase, thyroglobulin, cAMP, and DNA in human thyroid. *J Clin Endocrinol Metab* **43**: 466–469

Valenta LJ, Valenta V, Wang CA, Vickery AL, Caulfield J and Maloof F (1973) Subcellular distribution of peroxidase activity in human thyroid tissue. *J Clin Endocrinol Metab* **37**: 560–569

Yamashita H, Noguchi S, Murakami N, Adachi M and Maruta J (1993) Immunohistological differentiation of benign thyroid follicular cell tumors from malignant ones: Usefulness of anti-peroxidase and JT-95 antibodies. *Acta Pathol Jpn* **43**: 670–673

 *© 2001 Cancer Research Campaign*
